# How to Fabricate Hyaluronic Acid for Ocular Drug Delivery

**DOI:** 10.3390/pharmaceutics16121604

**Published:** 2024-12-16

**Authors:** Martha Kim, Mi-Young Jung, Do-Yeon Lee, So Min Ahn, Gyeong Min Lee, Choul Yong Park

**Affiliations:** 1 Department of Ophthalmology, Dongguk University Ilsan Hospital, Goyang 10326, Republic of Korea; ragi22@hanmail.net (M.K.); myjung202@gmail.com (M.-Y.J.); dyl120296@gmail.com (D.-Y.L.); ahnsomin808@gmail.com (S.M.A.); arips88@gmail.com (G.M.L.); 2 Department of Ophthalmology, Samsung Medical Center, Sungkyunkwan University School of Medicine, 81, Irwon-ro, Gangnam-gu, Seoul 06351, Republic of Korea

**Keywords:** hyaluronic acid, ocular disease, drug delivery, fabrication, modification, eye

## Abstract

This review aims to examine existing research on the development of ocular drug delivery devices utilizing hyaluronic acid (HA). Renowned for its exceptional biocompatibility, viscoelastic properties, and ability to enhance drug bioavailability, HA is a naturally occurring biopolymer. The review discussed specific mechanisms by which HA enhances drug delivery, including prolonging drug residence time on ocular surfaces, facilitating controlled drug release, and improving drug penetration through ocular tissues. By focusing on these unique functionalities, this review highlights the potential of HA-based systems to revolutionize ocular treatment. Various fabrication techniques for HA-based ocular drug delivery systems, including hydrogels, nanoparticles, and microneedles, are discussed, highlighting their respective advantages and limitations. Additionally, this review explores the clinical applications of HA-based devices in treating a range of ocular diseases, such as dry eye syndrome, glaucoma, retinal disorders, and ocular infections. By comparing the efficacy and safety profiles of these devices with traditional ocular drug delivery methods, this review aims to provide a comprehensive understanding of the potential benefits and challenges associated with HA-based systems. Moreover, this review discusses current limitations and future directions in the field, such as the need for standardized fabrication protocols, long-term biocompatibility studies, and large-scale clinical trials. The insights and advancements presented in this review aim to guide future research and development efforts, ultimately enhancing the effectiveness of ocular drug delivery and improving patient outcomes.

## 1. General Biology of Hyaluronic Acid (HA)

### 1.1. Natural Biosynthesis of HA

Hyaluronic acid (HA) is a naturally occurring linear polysaccharide composed of repeating disaccharide units of D-glucuronic acid and N-acetyl-D-glucosamine [1]. It is widely distributed throughout connective, epithelial, and neural tissues, where it plays crucial roles in cell proliferation, migration, and tissue hydration [1,2,3]. The biosynthesis of HA is catalyzed by enzymes known as hyaluronan synthases (HASs) [4,5]. In vertebrates, there are three main types of HAS: HAS1, HAS2, and HAS3 [6]. These enzymes, integral membrane proteins, facilitate the polymerization of HA by alternating the addition of glucuronic acid and N-acetylglucosamine to the growing polysaccharide chain [7,8]. The substrates for HA synthesis, UDP-glucuronic acid and UDP-N-acetylglucosamine, are derived from glucose metabolism [9]. The availability of these substrates is a key regulatory point in HA production [8]. Although HAS is located in the plasma membrane, the newly synthesized HA chain by HAS is directly extruded into the extracellular space [10]. Each HAS isoform possesses unique kinetic properties and distinct tissue distributions, which contribute to the regulation of HA molecular weight and its production rate [5,6,11]. The various HAS isoforms produce HA that varies in molecular weight. HAS1 and HAS3 generate HA polymers with molecular weights ranging from 2 × 10^5^ to 2 × 10^6^ Da, whereas HAS2 synthesizes HA polymers with a molecular weight exceeding 2 × 10^6^ Da [12]. Previous studies have indicated that the HAS2 isoform plays a crucial role in HA synthesis. The deletion of the HAS2 gene is lethal because knockout mice lacking HAS2 die on embryonic day [13].

HA synthesis is controlled by various factors, with the expression levels of HAS genes being regulated by different cytokines, growth factors, and cellular stress conditions [1]. For example, transforming growth factor-beta (TGF-β), epidermal growth factor (EGF), and platelet-derived growth factor (PDGF) are known to enhance HAS expression [14]. HAS can also undergo post-translational modifications, such as phosphorylation, which may influence their activity and stability [8]. Additionally, the degradation products of HA, generated by hyaluronidases, can influence HAS activity through a feedback mechanism. Typically, Buhren et al. reported that high-molecular-weight HA (491 KDa) tends to inhibit HAS activity, whereas low-molecular-weight HA (134 KDa) may stimulate it [12]. Under inflammatory conditions, low-molecular-weight HA (less than 100 KDa) synthesis by HAS is increased [15]. Beyond vertebrate systems, HA is also produced by certain microbial species [4,8,16]. Streptococcus species are the most commonly used microorganisms for industrial HA production [4]. These bacteria have their own HAS enzymes that function similarly to vertebrate HAS but with different regulatory mechanisms [8].

Recent advancements in synthetic biology have enabled the heterologous expression of HAS in non-pathogenic microbial hosts, such as *Bacillus subtilis* and *Escherichia coli* [16,17,18]. These genetically engineered strains can also produce HA with high yield and purity, presenting a safer and more sustainable alternative to traditional methods of extraction from animal tissues. To utilize HA in ophthalmology, it is essential to produce high-molecular-weight, high-purity HA. Therefore, recent manufacturing technologies that yield high amounts of high-purity HA are highly beneficial for developing HA-based ocular drug delivery platforms.

### 1.2. Biologic Function of HA

HA naturally plays several crucial roles in the body [1,7,19]. It exists in its sodium salt form, sodium hyaluronate, and is present in various soft connective tissues including the skin, the lungs, the kidneys, the brain, and muscles [19,20,21]. It plays fundamental roles in regulating tissue hydration, water transport, maintaining the viscoelasticity of connective tissues, facilitating the assembly of proteoglycans in the extracellular matrix, and acting as a joint lubricant to reduce friction and ensure smooth movement [19,22]. HA contributes to wound healing by controlling inflammation and directing blood flow to damaged tissues. It plays several receptor-mediated roles during the healing process, including cell detachment, mitosis, migration, tumor development, metastasis, and inflammation [23,24].

HA is involved in a cell signaling pathway that is essential for tissue repair and regeneration [7,19,24,25]. HA binds to specific cell surface receptors, such as cluster-determined 44 (CD44), receptor for hyaluronate-mediated motility (RHAMM), HA receptor for endocytosis (HARE), and lymphatic vessel endothelial hyaluronan receptor-1 (LYVE-1), activating intracellular signaling cascades that influence cell proliferation, survival, and motility [3,21,24]. HA can also modulate immune responses by interacting with immune cells [21,26]. Components of the HA signaling pathway, including HA synthases and hyaluronidases, independently contribute to tumor growth, metastasis, and angiogenesis, making them targets for cancer therapies [27,28].

The biological effects of HA are distinct from other biologically active molecules and are influenced by its molecular weight [1,4,15,20,29,30]. There is evidence that high-molecular-weight (HMW) HA can cluster more receptors on the cell membrane, whereas low-molecular-weight (LMW) HA does not possess the same ability to aggregate cell membrane receptors, resulting in different signaling outcomes compared to HMW HA in the same cells [4,15,31,32].

### 1.3. Degradation of HA

The human body contains 15 g of HA, of which one-third is turned over daily [1,7,33]. The half-life of HA in human tissues varies from three to five minutes in blood to approximately 70 days in the vitreous body of the eye, the site of slowest HA turnover. HA is degraded through two primary mechanisms [1,7]. One involves the enzymatic action of hyaluronidases (HYALs) while the other, non-specific, results from oxidative damage by reactive oxygen species (ROS). Under normal conditions, HA degradation is rapid, producing di- or tetra-saccharides that are further broken down into H_2_O and CO_2_, preventing the accumulation of harmful HA fragments in the tissue. However, small or intermediate-sized HA fragments accumulate under inflammatory conditions, which exacerbates inflammation [15].

The human genome includes six identified HYAL-related gene sequences: HYAL-1, HYAL-2, and HYAL-3 genes are clustered on chromosome 3p21.3; HYAL-4, PH20 genes, and the HYAL-P1 pseudogene are located on chromosome 7p31.3 [1,7,15,34,35]. These genes are implicated in the production of HYAL enzymes, involved in HA degradation. Although six HYAL genes are identified in human somatic tissue, only HYAL-1 and HYAL-2 are significantly expressed. HYAL-2, anchored to the cell membrane, cleaves high-molecular-weight HA into intermediate-sized fragments. Conversely, HYAL-1 mainly functions within lysosomes and collaborates with HYAL-2 to further degrade these fragments into tetra-saccharides [1,35].

Approximately 30% of HA degradation occurs within tissues such as the skin or joints [7]. The remaining roughly 70% of HA undergoes systemic catabolism, being transported to the lymph nodes via the lymphatic system where it is internalized and broken down by endothelial cells of the lymphatic vessels [7]. Furthermore, a very small fraction of HA enters the blood vessels and reaches the liver, where it is degraded by hepatic endothelial cells [7].

The degradation caused by hyaluronidase can be a hindrance to HA-based ocular drug delivery systems. To overcome this drawback, cross-linking or enzyme conjugation can be utilized. For instance, Kim et al. [36] confirmed the sustained release of drugs following subcutaneous injection using a hyaluronidase inhibitor-incorporated HA hydrogel cross-linked with 1,4-butanediol diglycidyl ether.

### 1.4. Manufacturing Commercial HA

In 1976, Pharmacia began manufacturing and marketing high-molecular-weight HA (HMW HA) under the trade name Healon for joint pain relief [37]. During that period, HA was extracted from animal tissues such as rooster combs and human umbilical cords, which posed risks of contamination and allergic reactions [35]. Currently, HA is mainly produced through microbial fermentation, using bacteria like *Streptococcus.* Initially, Streptococci strains A and C were utilized for HA production, whereas today, many commercial products like Restylane^®^ by Q-med AB and Juvederm^®^ by Allergan are derived from *Streptococcus equi* [1]. Biotechnological advances have facilitated the use of genetically engineered microorganisms for HA production, enhancing control over its molecular weight and purity [16,18,38]. This method proves cost-effective and scalable for large-scale production but necessitates endotoxin and bacterial contaminant purification. The process, advantages, and disadvantages of each method are detailed in Table 1.

### 1.5. Chemical Modification of HA

Each disaccharide unit of HA comprises four hydroxyl groups, one amide group, and one carboxyl group; these components can be modified to alter hydrophobicity and biological activity [39,40]. Such modifications are generally achieved through either chemical conjugation or radical polymerization, such as cross-linking [39,40]. Conjugation involves grafting a monofunctional molecule onto a single HA chain through a covalent bond, whereas cross-linking uses polyfunctional compounds to connect multiple HA chains via several covalent bonds, aiming to substantially alter the mechanical, rheological, and swelling properties of HA [40,41]. Although HA is highly hydrophilic and water-soluble, cross-linking can reduce its solubility, making it appropriate for various medical applications including drug carriers [25,40].

Chemical modifications of HA enable its transformation into various physical forms, such as viscoelastic solutions, hydrogels, fibers, flexible sheets, sponges, meshes, nanoparticles, and microparticles, vigorously applicable in both clinical and preclinical settings [42]. Chemically modified HA or HA derivatives present advantages over pure HA, including delayed dissolution in water, enhanced degradation resistance, and amphiphilic properties that facilitate drug encapsulation and delivery [28,43]. Hydrophobic modifications, such as esterification and amidation, enhance drug loading and provide sustained release. Additionally, cross-linking with agents like glutaraldehyde or photo-reactive groups can create HA hydrogels with controlled drug release properties [28].

Among chemical modifications, thiolated HA increases mucoadhesion properties, which extends the residence time on mucosal surfaces such as gastrointestinal, vaginal, buccal, and ocular tissues. When drugs are incorporated with a thiolated HA delivery system, sustained and localized drug release is achieved [39,44]. Kafedjiiski et al. showed a 6.5-fold prolonged adhesion time of thiolated HA comparing with HA [45].

Not only is grafting polymers like polyethylene glycol (PEG) or peptides onto HA feasible, but also it enhances the stability, biocompatibility, and targeting efficiency of HA [46,47]. Moreover, ionic modifications, including cationization and anionic modification, can enhance tissue interaction and drug retention [48,49]. These enhancements optimize HA’s drug delivery properties and improve therapeutic efficacy.

## 2. HA in Drug Delivery

**Figure 1:**
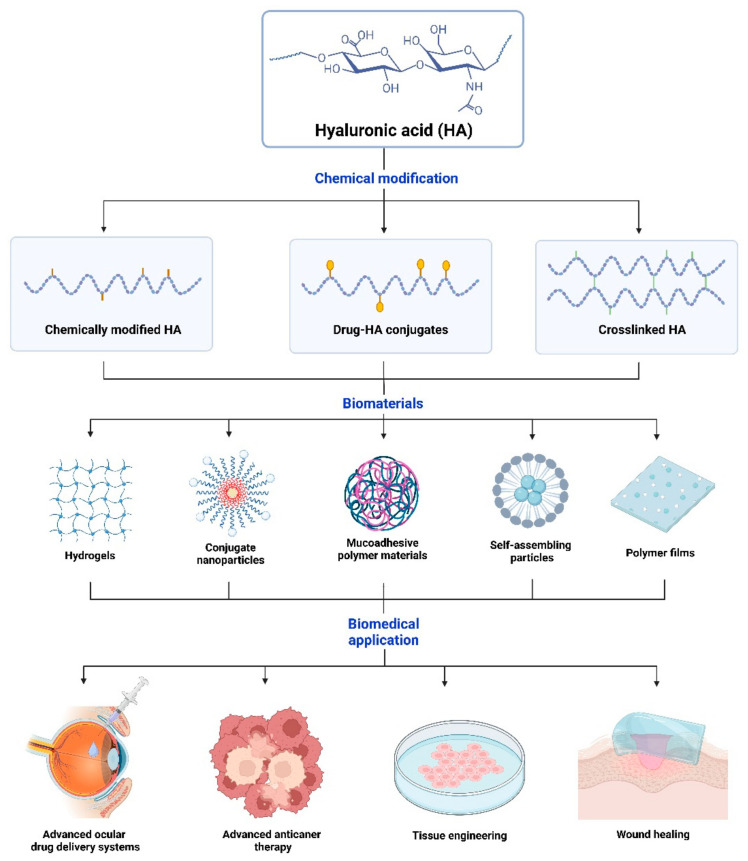
HA can be fabricated into various biomaterial forms through different chemical modification processes to optimize drug delivery characteristics for target tissues. By improving the properties of HA through various chemical modifications, it can be processed into different polymer forms such as hydrogels, nanoparticles, and films, making it applicable to a wide range of clinical conditions. Created in https://BioRender.com.

HA has advantages in targeted drug delivery due to its biodegradability, biocompatibility, and binding capacity to cell surface receptors [3,50,51]. It can be engineered as a carrier, forming conjugates with various drugs to enable controlled release and targeted effects (Figure 1) [19,28,37,52]. Conjugating active ingredients to HA results in a prodrug form that exhibits enhanced physicochemical properties, shelf life, stability, therapeutic efficacy, and safety compared to free drugs [28,39,40,48]. Upon drug release, therapeutic actions occur when the chemical bonds between the active ingredients and HA are catalyzed in the biological system, such as through enzyme- or pH-triggered cleavage, ideally at target sites [28,39].

HA is valuable in the manufacturing of controlled release or targeted drug delivery systems due to its excellent biocompatible gelation properties [28]. It can be compounded with a wide range of active ingredients for topical or intravenous applications [28]. Although HA is used in transdermal drug delivery, its high molecular weight hinders its ability to penetrate the stratum corneum [53]. This limitation can be mitigated by using HA nanoparticles, which efficiently deliver drugs to the dermis. Typically, HA-based nanoparticles improve drug encapsulation and stability [54,55,56]. Additionally, HA-based nanoparticles in polymeric thin films serve as hybrid therapeutic systems for controlled release, effectively managing skin wounds with vitamin E [57].

Chitosan-based nanoparticles with HA have been shown to increase corneal retention time, nearly doubling the bioavailability of dexamethasone [58,59]. These nanoparticles are also effective in gene delivery, ensuring efficient transfer without compromising cell viability. HA-modified liposomes are promising as drug carriers, enhancing drug stability in the bloodstream, extending drug half-life, reducing toxicity, improving tissue absorption and barrier permeability, and supporting sustained or controlled release. Collectively, these attributes enhance therapeutic efficacy through synergistic actions [60,61].

HA is predominantly used in cancer therapy for drug delivery [62]. It exhibits a strong affinity for CD44 receptors, prevalently expressed on numerous cancer cells [63]. This mechanism enables HA-based drug delivery systems to selectively accumulate in tumor tissues, thus enhancing the effectiveness of therapeutics and reducing adverse effects on healthy tissues [64]. HA can be manipulated into nanoparticles, micelles, or liposomes to encase chemotherapeutic drugs [65]. These carriers safeguard the drugs from degradation in the bloodstream, enhance their solubility, and ensure controlled release at the tumor site. HA-based systems can also co-deliver chemotherapeutic agents and immunomodulators, generating a synergistic effect that boosts the overall anticancer activity [65]. This strategy can assist in overcoming drug resistance and improving patient outcomes.

HA can be used to deliver photosensitizers for photodynamic therapy, a treatment that uses light to activate the drug and kill cancer cells [66,67]. HA-based systems ensure the selective accumulation of photosensitizers in tumor tissues, thus minimizing damage to surrounding healthy tissues. Tumor tissues often have slightly acidic environments compared to normal tissues. As HA is highly sensitive to pH changes, it degrades via hydrolysis when the pH falls below 4 or rises above 11 [68]. HA-based drug delivery systems can thus be engineered to release drugs in response to this pH difference, ensuring specific drug release at the tumor site. Certain enzymes are overexpressed in tumor tissues. HA can be modified to release the drug in the presence of these enzymes, offering a targeted and controlled release mechanism [67].

## 3. Limitations of Current Ocular Drug Delivery

**Figure 2:**
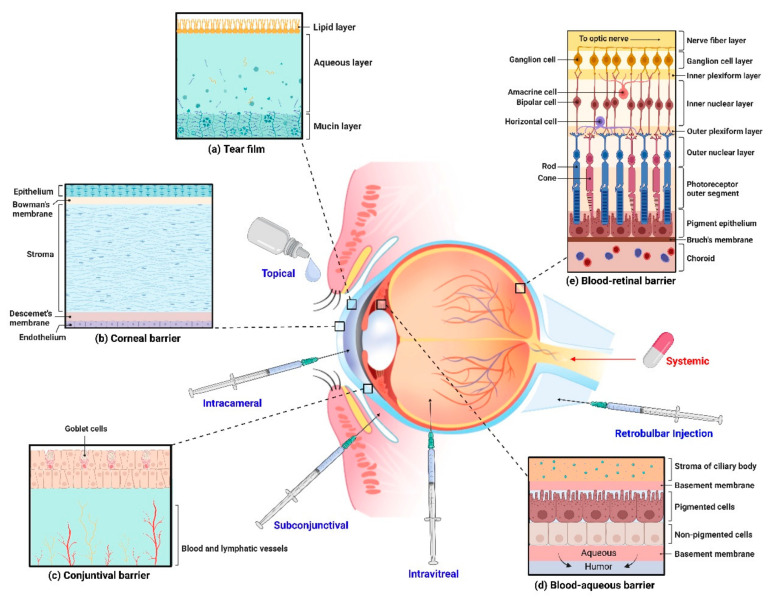
The eye presents several drug delivery barriers (tear film barrier, conjunctiva barrier, corneal barrier, blood–aqueous barrier, and blood–retinal barrier), requiring various drug delivery methods (eye drops, oral medications, and invasive injections) to address these challenges. Even for drugs that pass through the significant barrier of tears, the corneal barrier remains the primary obstacle preventing most eye drops from penetrating to the target tissues within the eye. For effective drug delivery, the drug must be designed to traverse both the hydrophobic corneal epithelium and the hydrophilic corneal stroma. Additionally, drugs that enter the aqueous humor are continuously diluted and removed by aqueous humor flow, resulting in very little drug reaching the vitreous and retina. Therefore, treating retinal diseases often involves direct injection of the drug into the vitreous cavity using a needle, a method that is invasive and carries the risk of pain and complications. Created in https://BioRender.com.

More than 90% of ophthalmic drugs are administered as eye drops or ointments, which are effective for treating corneal and conjunctival diseases. However, their intraocular penetration and bioavailability are limited by physiological barriers such as nasolacrimal drainage, lacrimation, and blinking, as well as anatomical barriers (Figure 2) [69,70].

The eye’s inferior cul-de-sac can retain approximately 30 μL of fluid [71], with excess drops being expelled after the initial blink. The tear film is replenished every five minutes, facilitating the removal of unabsorbed drugs [72]. The cornea’s surface comprises five to six epithelial layers with tight junctions that impede paracellular drug transport [72]. Hydrophobic drugs face challenges in penetrating the hydrophilic corneal stroma to reach the anterior chamber [69,73]. Although scleral penetration is more feasible, the conjunctiva, similar to the corneal epithelium, serves as a barrier to hydrophilic drugs [74]. As a result, less than 5% of topical ocular medication is absorbed due to these barriers [69].

Topical drug delivery is often ineffective for treating certain ocular diseases such as age-related macular degeneration (AMD). Patients with exudative AMD necessitate frequent intraocular injections of anti-VEGF agents, high-molecular-weight monoclonal antibodies that fail to penetrate the cornea and sclera topically [75]. These injections pose risks of infection and entail high costs due to the need for repeated treatments. To address these issues, intraocular implants have been developed, releasing corticosteroids for months to years with a single injection. Initially developed for retinal diseases due to market needs, these implants, which administer corticosteroids over extended periods with one injection, are now available. Prominent examples include Retisert (fluocinolone acetonide intravitreal implant, 0.59 mg, Bausch + Lomb) and Ozurdex (dexamethasone intravitreal implant, 0.7 mg, Allergan) [76,77]. Currently, anti-VEGF drug implants for long-term use are actively being developed [78].

In ophthalmology, the objective of developing drug delivery systems is to deliver medications efficiently in the least invasive manner possible. If an invasive method is necessary, the focus shifts towards prolonging the dosing interval using slow-release techniques. This approach helps reduce patient discomfort, lower socio-economic costs, and enhance treatment adherence. The features of HA-based ocular drug delivery compared to conventional eye drops are summarized in Table 2.

## 4. Potential Benefit with HA-Based Ocular Drug Delivery System

**Figure 3:**
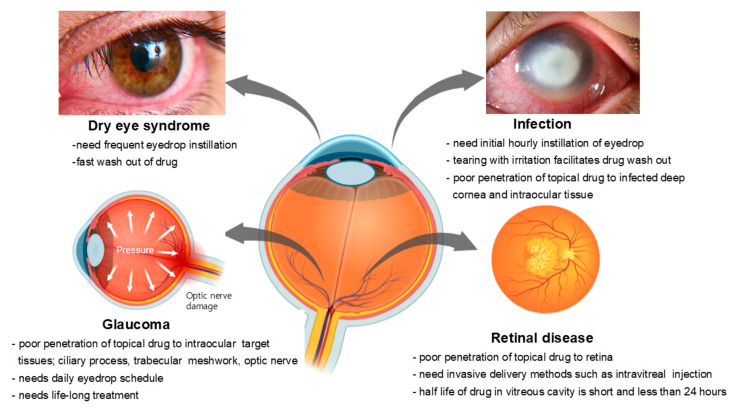
Various ocular diseases represent significant unmet needs in medical treatment. These diseases possess inherent challenges that are difficult to address with current therapies, depending on the type of disease. Created in https://BioRender.com.

Various ocular diseases can be addressed or managed with medication instead of surgical intervention. However, drug treatments for specific eye diseases are constrained by unique limitations for each condition, as depicted in Figure 3.

HA is extensively utilized in ophthalmology for various applications, including dry eye treatment, contact lens comfort agents, vitreous substitutes, corneal wound healing, drug delivery systems, and ophthalmic visco-surgical devices [3,37,50]. Its high biocompatibility is attributed to its natural presence in ocular tissues such as the cornea, aqueous humor, iris, lens, vitreous, and retina [3]. Furthermore, HA possesses mucoadhesive properties. It adheres to the corneal mucin layer through non-covalent bonds, where its acid groups interact with sialic acid in eye mucin [3,71,79]. Research has shown that increasing the molecular weight (MW) of HA or lowering the pH of the solution enhances its adhesion properties [1,7].

HA’s hydrophilic nature and extended chain length enable it to bind significant amounts of water, forming between 10 and 15 hydrogen bonds with HA’s disaccharide units [1]. This capacity to bind water is affected by both pH and concentration [1,7]. Consequently, HA-based drug delivery devices maintain comfort on the ocular surface. Furthermore, HA interacts with cells and tissues via specific receptors to perform various biological functions. CD44, extensively studied and utilized among these receptors, is present in retinal Müller glia microvilli, corneal epithelium, and endothelium [80]. The interaction of HA with CD44 involves complex multivalent interactions, with affinity increasing as the molecular weight of HA increases [30].

One of the key reasons HA is valuable in ocular drug delivery is due to its intrinsic effects, separate from the drugs it carries. HA positively influences corneal wound healing by promoting cell migration, enhancing repair responses, and reducing inflammatory responses [1,3]. Exposure to HA solutions decreases the expression of inflammatory cytokines IL-1β and MMP-9 in human corneal epithelial cells, while increasing the expression of repair factors CD44 and fibronectin [3].

Being biocompatible and biodegradable, HA is a safe option for ocular drug delivery systems [79]. HA-based systems can be engineered to provide controlled drug release, ensuring a steady and sustained therapeutic effect, which is particularly beneficial for chronic ocular diseases requiring long-term treatment [3]. HA can stabilize drugs, protecting them from degradation and maintaining their efficacy during the delivery process [28]. Moreover, HA can be used to form nanoparticles, which enhance intraocular permeation, prolong retention times, and improve drug stability [50,61]. These nanoparticles can be designed to carry both hydrophilic and lipophilic drugs, increasing the range of treatable conditions [27,51]. Considering these unique functionalities of HA, HA-based ocular drug delivery systems can offer innovative solutions that can improve patient outcomes.

## 5. Various HA Platforms for Ocular Drug Delivery Systems

**Figure 4:**
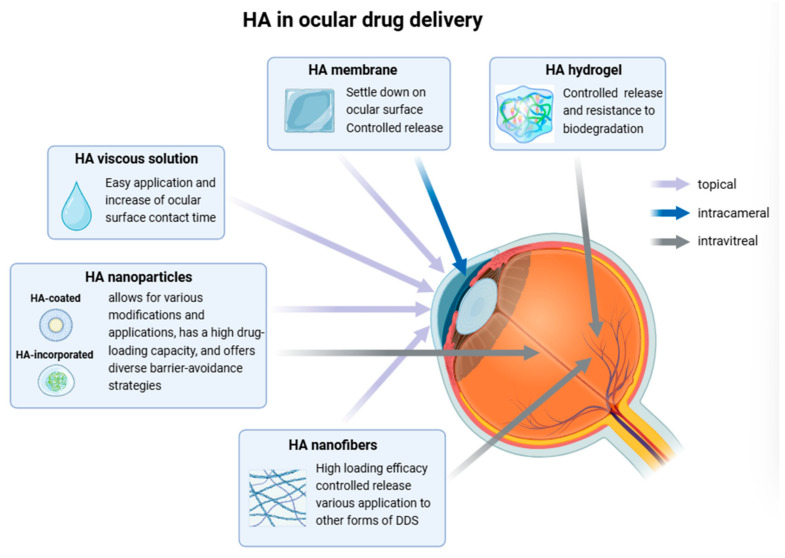
Fabrication of hyaluronic acid for various routes (topical, intracameral, and intravitreal) of ocular drug delivery. HA is designed as a drug delivery platform with appropriate forms, drug loading, and degradation characteristics tailored for specific ocular diseases. Created in https://BioRender.com.

In 1978, Healon was first employed in cataract surgery to protect the corneal endothelium [30]. The initial clinical use of hyaluronan eye drops for severe dry eyes was documented in 1982, when a 0.1% HA solution was derived from Healon syringes to manage keratoconjunctivitis sicca [30]. Since then, the application of HA in ophthalmology has expanded significantly. Currently, HA is widely used in eye drops and surgical adjuncts due to its viscoelastic properties, tissue hydration capabilities, and its promotion of ocular surface wound healing [3,37,79,80,81,82]. In addition to eye drops, HA is engineered into various forms of drug delivery platforms including nanofibers, nanoparticles, hydrogels, membranes, and films (Figure 4).

### 5.1. Simple Viscous Solution

Increasing the viscosity of eye drops by adding polymers enhances drug delivery by prolonging the retention time on the ocular surface. This method effectively prevents the drug from being washed away by tears, thus extending its delivery time into the eye [3]. HA uniquely binds to mucin on the ocular surface and to CD44 on eye surface cells, which increases the delivery time of co-administered drugs [3,7]. Additionally, the pseudoplastic fluid property of HA reduces the sensation of foreign bodies in viscous eye drops containing HA and ensures even distribution across the eye surface without impairing vision [1,37]. Consequently, HA is formulated into simple viscous solutions with various drugs. Remarkably, HA exhibits superior retention (retaining percent 30.4%, 12.7%, and 18.6% for HA, alginate, and chitosan, respectively) on the eye surface compared to chitosan or sodium alginate [83]. The retention of HA on the ocular surface is influenced by factors such as molecular weight, concentration, and intermolecular interactions between the drug and HA. It has been reported that a 0.3% HA solution retains two-fold longer than a 0.1% HA solution, and 1100 kDa HA exhibits almost four-fold greater retention than 250 kDa HA [84,85]. Battistini and colleagues developed viscous formulations containing HA and a timolol ionic conjugate [50]. These solutions increased retention time on the ocular surface by approximately 400% compared to the control. Moreover, the drug’s high affinity for the carboxylic groups of HA enables a slow-release profile for timolol, even in solution forms [50].

### 5.2. Nanofibers

Nanofibers are typically defined as filamentous structures with a diameter less than 1 µm. HA fibers can be produced using various methods, including wet spinning and electrospinning. The details of these manufacturing methods have been comprehensively reviewed [86,87]. Nanofiber scaffolds have effectively been used to deliver drugs, cells, and genes to body organs via diverse routes [87]. Thanks to their unique properties such as high loading efficiency, superior mechanical performance, controlled release, and chemical stability, they are instrumental in delivering large protein drugs, genetic materials, plasmid DNA, and therapeutic cells to targeted sites [86,87]. Nanofibers can be utilized as standalone finished products, or they can be further processed into diverse forms such as nanoparticles, meshes, sponges, and more [87]. Lokhande et al. [88] developed a biodegradable HA nanofiber insert for treating dry eyes. Nanofibers of HA in concentrations of 0.1%, 0.2%, and 0.5% combined with polyvinyl alcohol were created using the electrospinning technique. The nanofibers demonstrated drug loading efficiency ranging from 93% to 96% and exhibited a controlled drug release of 91.81% over 12 h [88]. Grimaudo et al. designed an ophthalmic insert made of HA nanofibers for the dual delivery of an antioxidant (ferulic acid and FA) and an antimicrobial peptide (ε-polylysine and ε-PL) [89]. The insert completely released ε-PL within 30 min and FA within 20 min under sink conditions.

HA nanofibers can also be fabricated to achieve sustainable drug release. Hosseini et al. demonstrated the effectiveness of biocompatible electrospun PVA/Chi/HA nanofibers for the sustained release of human growth hormone (hGH), with an initial burst release of 11% within the first 2 h, followed by 64% release after 48 h [90].

### 5.3. Nanoparticles

To control drug release, higher-molecular-weight HA with elongated chains offers advantages by creating significant entanglements that hinder water penetration into the nanoparticles. Furthermore, HA’s hydrophilicity allows more water molecules to surround the nanoparticles, providing an additional energy barrier against degradation [51].

Table 3 summarizes several recent studies utilizing HA nanoparticles for ocular drug delivery.

### 5.4. Hydrogels

The development of hydrogels using HA in the ophthalmic field has been primarily designed and researched for the slow release of HA on the ocular surface [99]. Hydrogels are complex polymeric networks with a three-dimensional architecture that allows them to absorb large amounts of water while maintaining structural integrity. HA-based hydrogels can be prepared using various methods such as polymerization, enzymatic cross-linking, condensation reactions, and click chemistry [7]. HA hydrogels can be directly cross-linked using agents such as glutaraldehyde, divinyl sulfone, bisepoxide, and carbodiimide [40].

HA can be engineered to form a hydrogel in situ. In situ gel is thermosensitive; it transitions from an aqueous solution at low temperatures to a gelatinous state as the temperature rises. The high viscosity of the gel prolongs ocular residence time, a beneficial property for ophthalmological applications. Zhu et al. [100] developed an in situ gel utilizing poly(N-isopropylacrylamide) and HA to deliver the antifungal agent ketoconazole to the ocular surface. The drug content of the prepared gels ranged from 91 to 96%, pH values were between 6.0 and 7.5, and the gelation temperature was 33 °C. In vitro release studies indicated that ketoconazole release from the in situ gels was moderate, without burst releases, and no irritant reactions were observed in the rabbit eye.

Chen et al. [101] developed an injectable HA hydrogel with 1,4-butanediol diglycidyl ether (BDDE) cross-linking. The anti-inflammatory agent, epigallocatechin gallate, was incorporated into the hydrogel. They assessed the efficacy of the injectable HA hydrogel to replace the vitreous humor in rabbits following normal vitreous aspiration. The results demonstrated prolonged degradation times up to 28 days and anti-inflammatory effects of the hydrogel postoperatively.

Table 4 summarizes several recent studies utilizing HA hydrogels for ocular drug delivery.

### 5.5. Membranes or Films

HA films or membranes offer several advantages over conventional formulations such as gels, ointments, and solutions. They are stable, long-lasting, and can enhance patient compliance. Research on HA-based films aims to overcome drug delivery barriers for various diseases. To be suitable for biomedical applications, HA films must have adequate mechanical strength when hydrated. Either physical or chemical cross-linking techniques can be applied to achieve this goal. Kim et al. developed a hyaluronic acid membrane cross-linked with 1,4-butanediol diglycidyl ether (BDDE) and incorporated moxifloxacin [106]. When inserted into the anterior chamber of the eye in a dry state, the membrane naturally hydrated and settled in. It was confirmed through in vivo experiments using rabbit eyes that moxifloxacin was released from the membrane into the anterior chamber of the eye at a concentration sufficient to inhibit *Pseudomonas aeruginosa* and *Staphylococcus aureus* for over 5 days after implantation [106]. The same researchers also developed cross-linked transparent HA membranes for treating ocular surface diseases [25]. Application of HA membranes for chemical corneal trauma and conjunctival surgery in rabbits revealed enhanced wound healing.

Palmitoyl esters of hyaluronan were developed into water-insoluble films to address hyaluronan’s solubility issues [107]. These films were solution-cast with smooth surfaces, uniform thickness, exhibited non-cytotoxicity, and did not adhere to cells, making them promising for biomedical applications such as tissue engineering and wound healing. Water-insoluble, free-standing films from lauroyl-modified HA were developed, with properties tunable by the degree of HA substitution. These films were homogeneous, mechanically strong, and flexible [108]. Hydrophobized or crosslinkable hyaluronan derivatives exhibited increased resistance to biodegradation. These films were safe, and their degradation could be tailored by the degree of HA substitution in vitro and in vivo. Ghezzi et al. [109] developed hydrophilic films composed of HA and polyvinyl alcohol. These films included levofloxacin and dexamethasone, demonstrating high drug loading capacity and controlled drug release over 6 h when applied to ex vivo porcine eyes.

## 6. Candidate Ocular Diseases for HA-Based Ocular Drug Delivery System

### 6.1. Dry Eye Syndrome

Dry eye syndrome is a complex disease caused by the disruption of homeostasis between the tear film, comprising lipid, aqueous, and mucin layers, and the ocular surface cells. With the increasing use of video displays, its prevalence is rising sharply, making it a significant segment of the ophthalmic treatment market [110].

HA is primarily used as the main component of artificial tears for treating dry eye syndrome. The beneficial effects of HA on ocular surface damage in dry eye syndrome have been confirmed through both animal models and human studies [37]. Besides its therapeutic properties, HA also serves as a delivery system for various drugs aimed at treating dry eye syndrome.

Lokhande et al. [88] employed the electrospinning method to create HA-containing nanofiber inserts. Nanofibers of HA at concentrations of 0.1%, 0.2%, and 0.5% combined with polyvinyl alcohol were produced using the electrospinning technique. They demonstrated that the HA loading capacity of the insert ranged from 93 to 96%, and HA was released in a controlled manner (91.81% over 12 h). Utilizing this HA nanofiber insert in a murine dry eye model, they noted an increase in tear film breakup time and a decrease in conjunctival inflammatory cytokines (TNF-α and IL-6) in the treated group compared to the control group.

HA itself can serve as an effective drug component in dry eye syndrome treatments. Ali et al. developed HA-imprinted soft contact lenses for delivering HA to the ocular surface [111].

### 6.2. Glaucoma

Glaucoma is a chronic neurodegenerative condition characterized by ganglion cell death, retinal nerve fiber layer atrophy, and optic nerve cupping. Persistent increases in intraocular pressure and diminished optic nerve blood flow are primary factors contributing to the disease [112]. Treatment strategies primarily target these abnormalities, requiring almost lifelong medication after diagnosis, which underscores the importance of patient compliance. Decreased compliance among elderly patients poses a significant challenge to successful treatment [112].

In treating glaucoma, reducing medication frequency to achieve therapeutic effects commonly enhances patient compliance. Enhancing drug penetration or increasing ocular surface residence time can improve drug availability, thereby decreasing the frequency of medication instillation. The incorporation of HA within the drug delivery system proves beneficial in this context. Alviset et al. [113] developed an advanced eye drop formulation by utilizing sodium hyaluronate as a thickener and polysorbate 80 as a surfactant. This combination facilitated the self-assembly of the system into micelles, which were used to load travoprost. This strategy not only improved the performance of the eye drops but also allowed for a reduced dose of travoprost, enhancing bioavailability in rabbits and minimizing side effects [113]. Furthermore, nano- or micro-structures formed through polymer self-assembly can significantly increase the viscosity of the solution, serving as effective carriers for controlled drug delivery. Battistini et al. employed an ionic complex of sodium hyaluronate and timolol. They observed a longer period of decreased intraocular pressure (10 h) and a more intense hypotensive activity compared to commercial timolol eye drops [50]. Wadhwa et al. developed HA-modified chitosan nanoparticles loaded with timolol and dorzolamide. They found the duration of drug effect increased from 8 h without HA modification to 12 h with HA modification [97].

When medication alone cannot sufficiently reduce intraocular pressure, surgical treatments such as trabeculectomy are performed to create a bypass for the aqueous humor in the anterior chamber, thereby achieving further reduction in intraocular pressure [112]. The formation of a filtering bleb is crucial to lowering intraocular pressure following trabeculectomy, which creates a bypass for aqueous humor into the subconjunctival space. 5-flurouracil inhibits fibroblast proliferation, thereby maintaining the filtering bleb in optimal condition. Bora et al. [103] developed a 5-fluorouracil releasing device using an HA-based poly(D, L-lactide-co-glycide) (PLGA) microparticle-laden composite hydrogel system. They observed sustained release of 5-fluorouracil for 15 days from the hydrogel in vitro.

### 6.3. Retinal Diseases

Retinal diseases are serious conditions that can lead to significant vision loss. The retina is a neural tissue membrane lining the inner wall of the eye, akin to the film in a camera. Medicines applied to the eye surface must penetrate the dense collagen layers of the cornea and sclera, diffuse through the aqueous humor, and stably traverse the vitreous humor to reach the retina at the back of the eye, particularly the macula [114].

Due to the blood–retinal barrier, which is similar to the blood–brain barrier, oral or intravenous drug administration is often ineffective for treating retinal diseases [78]. With an aging population, there is a rising incidence of age-related macular degeneration and retinal vascular disorders. Additionally, the risk of macular edema as a complication of cataract and other ophthalmic surgeries has increased. Consequently, drug delivery for retinal disease treatment is one of the most actively researched areas in ophthalmology. To enhance drug delivery to the retina efficiently, the most widely used method is injecting the drug into the eye. However, there is a need for technology that enables the injected drug to continuously release therapeutic concentrations over a long period within the eye. In this field, HA can be utilized [78].

Awwad et al. [115] manufactured a biodegradable protein drug carrier using a thermosensitive hydrogel, N-isopropylacrylamide, and acrylated HA for the sustained release of infliximab and bevacizumab to treat retinal diseases. They observed a zero-order release profile of the protein drugs from day 5 to day 50, with 43.3 ± 9.5% of the protein released by day 50.

Injectable hydrogels have the potential to encapsulate drug components and release them with sustained kinetics. HA is a popular component for fabricating injectable hydrogels. Yu et al. [116] developed an in situ hydrogel through catalyst-free chemical cross-linking between vinylsulfone-functionalized hyaluronic acid and thiolated dextran. Bevacizumab was administered into the vitreous cavity of rabbit eyes using this in situ hydrogel. They observed therapeutic concentrations of bevacizumab in rabbit eyes for at least 6 months. Egbu et al. [117] created two gel systems using HA to deliver infliximab. The first system involved 1% and 5% tyramine-substituted HA cross-linked enzymatically in the presence of infliximab (HA-Tyr). The second hydrogel comprised N-isopropylacrylamide chemically cross-linked with HA and infliximab INF incorporating 1 and 3% poly(ethylene glycol) diacrylate (PEGDA-HA-pNIPAAM). In a two-compartment in vitro outflow model of the human eye, infliximab release rates were 45.4 ± 0.3 and 24.9 ± 0.4% on day 9 for 5% HA-Tyr and 1% PEGDA-HA-pNIPAAM, respectively.

HA can be combined with nanotechnology to enhance drug delivery to the retina. Laradji et al. [56] developed HA-coated gold nanoparticles to topically deliver drugs to the retina. They observed that hyaluronic acid-coated gold nanoparticles showed a higher distribution (nearly double the concentration) in the posterior segment of the eye compared to uncoated gold nanoparticles. Silva et al. [92] explored a chitosan–HA nanoparticle system for delivering epoetin beta (EPOβ) to the posterior segment of the eye following topical application. They detected EPOβ in the retina for up to 21 days, demonstrating the efficacy of their nanoparticle system.

### 6.4. Ocular Infection

Ocular infections remain a significant cause of blindness in underdeveloped regions. Such infections include keratitis, scleritis, conjunctivitis, and endophthalmitis. If not diagnosed promptly and treated correctly, they can lead to severe outcomes such as corneal opacity, cataracts, or even endophthalmitis, which involves the entire eye and may result in its loss [118,119]. Consequently, hyaluronic acid (HA) has proven effective as a delivery system for antibiotics to the eye. HA-based drug delivery systems are being extensively researched for managing ocular infections by integrating antibiotic agents.

For patients with bacterial keratitis, symptoms such as discomfort, pain, and tearing can render antibiotic eye drops less effective. In such instances, a delivery platform that sustains antibiotic presence on the ocular surface over an extended period offers more benefits than eye drops. Xie et al. [120] developed a hyaluronic acid-based in situ punctal plug encapsulating drug-loaded microcapsules. Forming from liquid to solid upon in vivo injection, this punctal plug adapts to various lacrimal duct anatomies and avoids the issues typical of conventional punctal plugs. They observed that higher cross-linking density and microencapsulation of ofloxacin resulted in slower drug release compared to lower cross-linking and a simple mixture of ofloxacin.

Fungal keratitis, a particularly stubborn form of infectious keratitis, requires extended topical antifungal treatment. Zhu et al. [100] introduced a thermosensitive in situ forming gel formulation of ketoconazole, utilizing poly(N-isopropylacrylamide) and HA. This formulation enhances biocompatibility and ocular surface residence time while extending drug release, without causing irritation to rabbit eyes.

After cataract surgery, patients typically require the administration of antibiotic and steroid eye drops four to six times daily for several weeks. These medications prevent bacterial infections and diminish inflammation resulting from the surgery. However, administering eye drops frequently poses challenges for elderly patients. In this context, a slow-releasing drug delivery system utilizing HA presents a viable alternative. Kim et al. [106] developed a hyaluronic acid membrane cross-linked with BDDE that encapsulated moxifloxacin [106]. This delivery system aims to replace antibiotic eye drops used to prevent infections post-surgery and maintain therapeutic levels of antibiotics in the anterior chamber of the eye for a 5-day critical period. The system was placed in the anterior chamber in a dry state and naturally absorbed moisture from the aqueous humor. Its transparent nature minimized any side effects associated with light blockage, and it gradually decomposed, releasing the antibiotic progressively. It was verified through in vivo experiments on rabbit eyes that the membrane released moxifloxacin at a concentration adequate to inhibit *Pseudomonas aeruginosa* and *Staphylococcus aureus* for over 5 days after implantation. Ghezzi et al. [109] developed hydrophilic films composed of HA and polyvinyl alcohol for delivering levofloxacin and dexamethasone into the eye, observing controlled drug release over 6 h. However, to serve as an effective alternative to eye drops, it is crucial that the drug delivery is extended to at least the initial 5 days post-surgery, when infection risk is at its peak.

## 7. Current Limitations of HA Fabrication for Ocular Drug Delivery System

HA is a promising material for ocular drug delivery, though its fabrication and utilization in ocular diseases face several impediments. Stability issues arise due to HA’s susceptibility to degradation by hyaluronidase enzymes in the eye, potentially reducing its effectiveness over time [121]. This enzymatic breakdown poses a challenge in maintaining sustained drug release. Moreover, while HA’s viscoelastic properties are beneficial for certain applications, they can complicate the formulation process [7]. High viscosity may impede uniform drug distribution and ease of administration. The biological activity and interaction of HA with ocular tissues largely depend on its molecular weight [30]. However, achieving consistent molecular weight and properties throughout the delivery system can be difficult, leading to variability in therapeutic outcomes.

As expected, HA’s large molecular size can limit its ability to penetrate ocular barriers, such as the corneal epithelium and blood–retinal barrier, thereby reducing its effectiveness in delivering drugs to deeper ocular tissues [69,78]. Developing stable and effective HA-based formulations requires detailed consideration of factors such as pH, ionic strength, and the presence of other excipients, which can profoundly affect HA’s stability, drug loading capacity, and release profile [52,71]. Furthermore, producing HA-based drug delivery systems at a commercial scale while maintaining quality and consistency presents significant challenges due to HA’s complex structure and sensitivity to environmental conditions [1,7]. Moreover, producing high-quality HA, especially with specific molecular weights and purity levels, can be costly, posing a barrier to the widespread adoption and development of HA-based ocular drug delivery systems [4,23,84].

The risk of immune reaction against HA is another limitation to be addressed. Various inflammatory reactions such as cellulitis, delayed immune responses, and allergic reactions including urticaria and angioedema have been reported following HA injection into the skin as a form of dermal filler [15]. It has been noted that CD44 can be upregulated by immune cells in certain pathological or inflamed conditions [122].

## 8. Economic Implications and Commercialization Potential of HA-Based Ocular Drug Delivery System

The economic implications of HA-based ocular drug delivery systems are promising, driven by the increasing prevalence of ocular diseases and the need for more effective treatments, especially with aging populations [3]. The global ocular drug delivery market is projected to grow significantly, with an annual growth rate of 8.4%, as reported in the Ophthalmic Drugs Market Size, Share & Trends Report, 2030. Within this market, HA-based systems offer advantages such as improved drug stability, prolonged retention time, and targeted tissue delivery. These systems aim to reduce the frequency of dosing and enhance patient compliance, potentially leading to cost savings in healthcare. Additionally, recent advances in commercial HA production technology have made HA-based ocular drug delivery systems more cost-effective.

The commercialization potential of HA-based ocular drug delivery systems is substantial, given their ability to address unmet medical needs in treating chronic ocular diseases such as glaucoma, age-related macular degeneration, and diabetic retinopathy. The biocompatibility and natural origin of HA make it an attractive option for developing safer ocular drug delivery systems. With ongoing research and development, HA-based ocular drug delivery systems have the potential to become a key player in the ocular drug delivery market, offering innovative solutions that improve patient outcomes and drive economic growth in the pharmaceutical industry.

However, currently there are no commercial HA-based ocular drug delivery systems available for clinical use [123]. The development and commercialization of HA-based systems must comply with regulatory requirements set by government agencies such as the FDA. These regulations ensure the safety, efficacy, and quality of HA-based products.

## 9. Conclusions and Future Perspectives

HA is a valuable material for ocular drug delivery, proven safe through its longstanding use in artificial tears. Its mucoadhesive properties, ability to interact with CD44, and inherent lubricating and wound healing effects distinguish it from other biomaterials. Although HA’s application in ocular drug delivery has not been widely commercialized, some studies and products have utilized HA-coated contact lenses to enhance comfort on the ocular surface. However, this approach has not been extensively developed for broader drug delivery applications [124].

For HA-based ocular drug delivery systems to successfully enter the market, they must be specifically designed to target the intended disease. The application method should be user-friendly for both clinicians and patients, ensure appropriate drug release kinetics for effective treatment, and be safe for long-term use. If inserted into the eye, the delivery device should not obstruct the field of vision. It should also exhibit excellent loading capacities and stability for hydrophilic and lipophilic drugs, as well as protein drugs often used in contemporary retinal disease treatments.

Future research into advanced HA derivatives with enhanced properties could further improve drug delivery efficiency and patient outcomes by offering better stability, increased drug loading capacity, and controlled release profiles. Conducting large-scale clinical trials is essential to establish the safety, efficacy, and long-term benefits of HA-based ocular drug delivery systems, providing the necessary data to support regulatory approval and commercialization. Exploring the integration of HA-based systems with smart drug delivery technologies, such as responsive hydrogels or nanoparticle systems, could offer more precise and personalized treatment options. Partnering with pharmaceutical companies can accelerate the development and commercialization of HA-based ocular drug delivery systems, leveraging their expertise, resources, and market presence.

Traditional eye drop treatment has been used by humanity for thousands of years to manage ocular diseases. While it beneficially dilutes risk factors on the eye surface, the rapidly advancing knowledge and technology in biomaterials engineering indicate that traditional eye drops will soon be surpassed by innovative drug delivery devices. HA-based drug delivery is expected to evolve rapidly.

## Figures and Tables

**Table 1:** Manufacturing methods of commercial-scale hyaluronic acid.

	In Vitro Production	Bacterial Production	Extraction from Animal Tissue
Process	Utilizes enzymes derived from *Streptococcus pyogenes*, *Pasteurella multocida*, and *Lactococcus lactis* to synthesize HA in a controlled laboratory environment.	Involves using various bacterial strains, including *Streptococcus*, *Enterococcus faecalis*, *Escherichia coli*, *Bacillus subtilis*, and *Lactococcus lactis.*	Isolates HA from various animal sources, including rooster comb, human umbilical cord, bovine synovial fluid, and vitreous humor of cattle.
Advantage	Allows for the production of HA with precise molecular weight and purity.	This method is relatively inexpensive and scalable, making it ideal for large-scale production.	Provides an essential source of HA for specific applications.
Challenge	Endotoxins produced by bacteria and the cost of expensive growth media pose challenges.	Often contains significant amounts of endotoxins and elevated bacterial levels, requiring additional purification steps to remove these impurities.	The risk of viral contamination necessitates complex purification procedures. The harsh extraction process often results in poor yield and polydispersity of molecular weights.
Application	Ideal for pharmaceutical and biomedical applications due to its high quality.	Suitable for commercial and industrial purposes due to its cost-effectiveness and scalability.	Used in specific applications where animal-derived HA is preferred or required.

**Table 2:** Comparison of conventional eye drop therapy and HA-based ocular delivery system.

	Conventional Eye Drop Therapy	HA-Based Ocular Drug Delivery System
Drug loading capacity	Limited	High using nanotechnology and chemical modification
Ocular surface retention time	Short	Prolonged due to mucoadhesive property and cell receptor binding
Drug delivery efficiency to intraocular target	Moderate	High by increasing drug loading, retention, and penetration through ocular barriers
Target disease spectrum	Limited to ocular surface diseases and glaucoma	Broad including ocular surface diseases, glaucoma, and retinal diseases
Safety	Generally safe	Probably safe, but individual drug delivery systems need to be evaluated
Economic cost	Low	Moderate
Manufacturing	Easy	Complex

**Table 3:** HA-based nanoparticles for ocular drug delivery.

Nanoparticles	Role of HA	Purpose	Finding	Refs.
HA-based gold nanoparticles	Surface coating	Topically applied hyaluronic acid-coated gold nanoparticles as drug delivery vehicles to the posterior region of the eye	Hyaluronic acid-coated gold nanoparticles exhibit higher distribution in the posterior segment of the eye compared to uncoated gold nanoparticles	[54,56]
Ciprofloxacin-loaded zein/hyaluronic acid nanoparticles	Mucoadhesion	To enhance the topical delivery of antibiotics to the ocular surface	A high encapsulation efficiency was achieved, with the release profile characterized by an initial burst followed by a sustained release of ciprofloxacin over 24 h	[55]
Gelatin–epigallocatechin gallate nanoparticles (GEH NPs) with surface decoration by hyaluronic acid (HA)	Surface coating	To topically deliver anti-inflammatory medication for the treatment of dry eye syndrome	Large quantities of GEH NPs accumulated in the cytoplasm of ocular surface cells, demonstrating the nanoparticles’ efficacy in ocular medication delivery	[91]
Epoetin beta-containing chitosan–hyaluronic acid nanoparticles	Mucoadhesion	For topical delivery of epoetin beta to the retina	Identification of epoetin beta in the retina 12 h post-administration, with its presence still detectable on day 21	[92]
Hyaluronic acid-coated albumin nanoparticles	CD44-mediated interaction	Targeted Connexin43 mimetic peptide delivery to the retina	HA-coated albumin NPs showed enhanced in vitro cellular uptake and ex vivo retinal penetration through HA-CD44 receptor-mediated interactions	[93]
Hyaluronic acid–chitosan–latanoprost-linked nanoparticles	Mucoadhesion	Topical administration to deliver IOP-lowering drugs	Daily IOP reduction during the treatment period was 24% for plain latanoprost, 23% for Xalatan, and 29% for HA–chitosan–latanoprost-linked nanoparticles	[94]
DNA-filled hyaluronic-acid nanospheres	Drug encapsulation	To deliver therapeutic genes to the outer retina via intravitreal injection	Follow-up at 4 weeks showed widespread gene expression in the outer retina, with reduced expression still present at 8 weeks post-injection	[95]
Gelatin–epigallocatechin gallate (EGCG) nanoparticles surface-decorated with HA and possessing a positive surface charge	Surface coating	To deliver drugs topically or via subconjunctival injection to the posterior segment of the eye	Fluorescent signals from nanoparticles were observed in the posterior eyes following topical and subconjunctival applications	[96]
Hyaluronic acid-modified chitosan nanoparticles (CS-HA-NPs) loaded with timolol and dorzolamide	Mucoadehsion	Topical administration to deliver IOP-lowering drugs	A significant reduction in IOP levels was achieved using CS-HA-NPs compared to a plain solution of the drugs	[97]
Sperminated HA-functionalized nanoceria (Ce-sH NPs) with insulin-like growth factor 1 (IGF1)	Drug encapsulation	Topical delivery of IGF1 for the healing of corneal wound	IGF1 loaded Ce-sH NPs showed high drug entrapment and controlled release resulting in better wound healing effect in a corneal alkali burn animal model	[98]

**Table 4:** HA hydrogels for ocular drug delivery.

Hydrogel	Description	Purpose	Findings	References
Hyaluronic acid hydrogel with curcumin nanoparticles	Curcumin nanoparticles were prepared using β-cyclodextrin as the encapsulating agent, and were incorporated into the hyaluronic acid-based hydrogel matrix.	To enhance keratitis healing	Curcumin nanoparticles with β-cyclodextrin in a hyaluronic acid hydrogel significantly reduced the frequency of medication administration compared to conventional therapies, enhanced the quality of healed structures, and effectively treated ulcerative keratitis.	[102]
5-Flurouracil (5-FU) microencapsulation and impregnation in hyaluronic acid hydrogel	5-FU was encapsulated in poly (d,l-lactide-co-glycolide) (PLGA) microspheres for sustained release and impregnated into a hyaluronic acid hydrogel.	For the sustained delivery of 5-FU to prevent ocular fibrosis	The release rate of 5-FU was retarded for a period of 6 days, and the overall release period was extended by 2 days, indicating sustained release.	[103]
HA-based hydrogels to encapsulate mouse retinal progenitor cells (RPCs)	In three-dimensional HA hydrogels, RPCs maintained their undifferentiated state and readily formed neurospheres.	To deliver RPCs to the retina to restore retinal function	Hydrogels were completely degraded, and RPCs were evenly distributed in the subretinal space by week 3, expressing the mature photoreceptor marker recoverin.	[104]
Chemically modified HA hydrogel with latanoprost-loaded liposome	HA was first chemically modified using adipic dihydrazide or methacrylic anhydride, mixed with latanoprost-loaded liposomes, and then cross-linked to produce nanocomposite hydrogels.	For the sustained release of latanoprost through topical application	Composite hydrogels provided a longer sustained release of latanoprost compared to liposomes or hydrogels alone.	[105]

## Data Availability

The datasets generated during and/or analyzed during the current study are available from the corresponding author on reasonable request.
